# Digestibility, textural and sensory characteristics of cookies made from residues of enzyme-assisted aqueous extraction of soybeans

**DOI:** 10.1038/s41598-020-61179-9

**Published:** 2020-03-06

**Authors:** Yang Li, Yufan Sun, Mingming Zhong, Fengying Xie, Huan Wang, Liang Li, Baokun Qi, Shuang Zhang

**Affiliations:** 1 College of Food Science, Northeast Agricultural University, Harbin, Heilongjiang 150030 China; 2 National Research Center of Soybean Engineering and Technology, Harbin, 150030 China; 3 Harbin Institute of Food Industry, Harbin, 150030 China

**Keywords:** Dietary carbohydrates, Nutrition

## Abstract

Enzyme-assisted aqueous extraction residue (REAE) has a lower utilization rate as it is the “waste” produced after the enzyme-assisted aqueous extraction (EAE), but its nutritional value is high. To improve the development and utilization of REAE, in this study, cookies were made by adding REAE (0%, 10%, 20%, 30%, 40%, 50%) as a food additive to a small amount of flour. The AOAC method was used to identify the basic components of REAE, analyze its physical and chemical properties, and characterize the cookie structure change in terms of texture, disulfide bond, and thiol content. An *in vitro* simulation system and sensory evaluation mechanism were established to analyze the bioavailability and impact of quality. The results show that REAE is a potential food additive. With an increase in the REAE content, the cookies become lighter in color, the sweetness and fat content are reduced, the hardness is increased, and the digestibility and glycerin index are reduced. The change in texture is caused by the reduction of disulfide bonds in the dough. The cookies were ‘well accepted’ with up to 30% REAE. Therefore, the use of the appropriate amount of REAE as a new food additive will reduce the amount of starch added.

## Introduction

Health-oriented products, such as low-calorie and high-fiber products, are increasingly purchased by consumers. Cookies are popular because of their characteristic texture and taste, and starch is an important carbohydrate source that provides energy for humans^1^. However, excessive starch consumption adversely affects blood glucose and increases the risk of obesity and associated diseases, such as Type II diabetes^2^

Dietary fiber, especially soluble dietary fiber (SDF), can be dispersed in water and forms colloids in the intestine to slow down the postprandial blood glucose response. Grigelmo-Miguel and Martín-Belloso^3^ proposed that the value of insoluble dietary fiber (IDF)/SDF that ranged from 1 to 2.3 is high quality dietary fiber, while most natural dietary fiber have much more IDF than SDF; therefore, adding SDF may be beneficial as part of a calorie-reduced diet^4^. The digestibility of fiber-enriched cookies decreased the starch release rate and the glycemic index (GI)^5^. SDF slows starch hydrolysis, digestion and absorption in the human small intestine. Dietary fiber can serve as a functional ingredient to improve the physical and structural properties, oil holding capacity, texture, sensory characteristics, and shelf-life of foods^6^. Dietary fiber has also been reported to disrupt the structure of amylopectin and reduce the free water content in a system; thus, the gelatinization, viscosity and rheological properties of starch are markedly changed by adding dietary fiber^7,8^.

Okara is a nutritious by-product of soybean processing and a good source of dietary fiber. Approximately, 0.4 kg okara is produced from each 1 kg of processed soybean, such as soybean milk, tofu, and soybean oil. Most okara is discarded or used as an animal feed. Therefore, an acceptable application of okara could have economic benefits^9,10^. Lu, *et al*.^11^ made acceptable wheat flour products supplemented with okara powder, although they found that excess addition of okara would dilute the gluten content in the dough, which hurt the product’s texture^12^. To be commercially successful as an improved cookie product, it is important to increase the dietary fiber of the cookies while maintaining acceptable texture.

The residues from the enzyme-assisted aqueous extraction (EAE) of soybean is a form of okara that contains more SDF, and its edibility and applicability are higher than normal okara^13^. In the present study, high dietary fiber cookies supplemented with residues from EAE (REAE) were prepared, and then their physicochemical and sensory properties, and the digestibility of the cookies were investigated^14^.

## Materials and Methods

### Materials

Mature soybeans of the variety Nong-Ken #42 (Heilongjiang Academy of Agricultural Sciences, Harbin, Heilongjiang, China) were crushed using a twin-screw extruder (EV032, Evolum, Clextral, Firminy, France) at 60 °C with screw speed of 120 rpm. Spring wheat flour (Kerry Food Industry Co., Harbin, Heilongjiang, China), sugar, instantized milk powder, salt, unsalted butter and eggs were purchased from a local WalMart Inc. (Harbin, Heilongjiang, China). 2.4 L Alcalase (activity 1.2 × 10^5^ U/mL), pepsin (from porcine gastric mucosa, 2500 U/mg), trypsin (from porcine pancreas, 250 U/mg), α-amylase (from porcine pancreas, 45.5 U/mg), and amyloglucosidase (3.6 × 10^4^ U/mL) were purchased from Sigma-Aldrich (St. Louis, MO, USA). All other chemicals were of analytical grade and obtained from Wilmar Co. (Harbin, Heilongjiang, China).

### Residues preparation

Sample (200 g) was mixed with 1200 mL distilled water and 1 g of 2.4 L Alcalase and transferred into a 55 °C water bath. The mixture pH was adjusted to 9.0 using 1 M NaOH. After 3 h, the enzyme was inactivated by boiling the mixture at 100 °C for 10 min, and the mixture was centrifuged at 4500 g for 20 min at 4 °C (GL-21M, ThermoFisher, Shanghai, China). The residues was dried at 60 °C in a drying oven.

Residues from soymilk (RSM) was used as the control sample. Rinsed soybeans (100 g) were soaked in 300 mL distilled water for 24 h at 4 °C then drained, and soymilk was extracted in 700 mL distilled water at 20 °C using a blender (JYL-350A, Joyoung, Jinan, Shandong, China) for 3 min at the highest speed. The whole slurry was filtered through a filtering cloth (400 mesh, Tongxing, Harbin, Heilongjiang, China) and the residues were collected.

Using AOAC methods, the compositions of REAE and residues from soymilk were determined, including moisture (AOAC 984.20), ash (AOAC 942.05), crude protein (AOAC 988.05) (Kjeldahl factor of 6.35) and crude fat (AOAC 920.39), and the total dietary fiber along with soluble and insoluble content were determined using the AACC 32-07 method.

### Cookie preparation

The cookie preparation was modified from Park, *et al*.^15^: 10 g sugar, 30 g skim milk powder and 0.5 g salt were well mixed. After addition of 30 g butter, the mixture was beaten with an electric mixer (JYL-F700, Joyoung) for 2 min. Whole egg (80 g) was added with another 1 min of beating. Sample flour (100 g, flour replaced with 0, 10, 20, 30, 40, or 50% REAE) was added. The dough was sheeted using a rolling pin with 3 mm rubber rings at both ends to obtain ~3 mm thick sheets. The sheets were cut using a round cookie cutter (45 mm diameter). The dough was placed on an ungreased aluminum baking tray, and the cookies were baked at 180 °C for 15 min. After baking, the cookies were cooled at 25 °C on a cooling wire.

### *In vitro* digestibility and starch digestibility measurement

The starch content in the cookies was measured using the Total Starch Assay Kit (K-TSTA, Megazyme, Bray, Ireland). The digestibility was done using an *in vitro* digestibility model of Smith, *et al*.^16^ as follows: the cookies were dispersed in 10 mL of simulated gastric fluid (SGF, pH 2.5, 0.9 mM NaH_2_PO_4_, 3 mM CaCl_2_, 0.1 M HCl, 0.15 M NaCl, 16 mM KCl), and 3.6% (w/v) pepsin was added and incubated with shaking for 60 min at 37 °C at 170 rpm. After adjusting the SGF to pH 6.5 with 37 °C Krebs-Ringer buffer (0.7 mM Na_2_HPO_4_, 0.49 mM MgCl_2_, 4.56 mM KCl, 1.5 mM NaH_2_PO_4_, 54.5 mM NaCl, and 80.4 mM NaHCO_3_), 6 mg of trypsin (250 U/mg), 65.9 mg of α-amylase (45.5 U/mL) and 40 μL of amyloglucosidase (1000 U/mL) were added, and incubated at 37 °C at 170 rpm for 0, 10, 20, 30, 60, 90, 120, 150,180, and 240 min. TCA (10%, 10 mL) was added to stop the reaction. The released glucose and starch digestibility was measured using the method of Englyst, *et al*.^17^: hydrolysate mixed with equal volume of DNS reagent were heated in a boiling water bath for 15 min, and absorbance at 575 nm was measured at 25 °C using the spectrophotometer with 0–0.5 mg/mL glucose as the glucose standard. The starch digestion rate is expressed as the percentage of total starch (TS) hydrolyzed at different times with each sample having 150 mg TS. The hydrolysate (0.5 mL) removed at 20 and 120 min of intestinal digestion (G_20_ and G_120_, respectively) was used to calculated the rapidly digestible starch (RDS) and slowly digestible starch (SDS), respectively, and the resistant starch (RS) was what remained^18^. Glycemic index (GI) and hydrolysis index (HI) were calculated using the starch digestion rate curve (glucose release)^19^. HI was the area under the curve for the experimental sample divided by the area under the curve of the control sample (white bread (water and flour)), and GI = 39.71 + 0.549 HI.

### Texture analysis

The textural properties of cookies doughs and cookies were measured using a Texture Analyzer (TA-XT2i, Stable Micro Systems, Godalming, UK). The dough samples were formed into cubes (~1 cm3), and their hardness, springiness and chewiness were measured using a flat cylindrical probe (P/36 R, 2 cm in diameter) and the pre-test, test and post-test speeds were set at 2.0, 1.0 and 1.0 mm/s, respectively, with the compression distance being 5 mm, essentially full compression^20^. The texture of cookies was determined with a spherical probe (P/0.25 S, 2.5 mm in diameter) and the pre-test, test, and post-test speeds were 1.5, 2, and 10 mm/s, respectively, with the compression distance being 3 mm with the cookies on a hard surface. Hardness (as the fracture force) of cookies was set at a trigger force of 25 g using a load cell of 50 kg, and was the maximum value of the force (g) at the cookies’ breaking point^21^. Tests were done in triplicate.

The diameter and thickness of cookies were measured with a Vernier caliper (SMIEC, Shanghai, China) which could measure 0.02 mm increments. The diameter of cookies was measured by putting six cookies edge to edge, and again after rotating each cookie 90°; thickness of six cookies stacked on top of each other in different orders were measured; then the average diameter and thickness were calculated. The spread factor was calculated from the ratio of the average diameter divided by the average thickness. Weights were measured using an electronic weighing balance (ES1200 Deante, Shanghai, China)^22^.

### Determination of the disulfide content in dough

Dough samples were freeze dried (FD5-3, Siemon Co., Ltd., Watertown, CT, USA) and milled into powder using a multipurpose electric grinder (HK-230, Kaichuang Tonghe Co., Beijing, China), and sieved through a 150 mesh screen. The free sulfhydryl-groups (–SH–) were determined^23^ as follows: 150 mg dough powder suspended in 1 mL Tris–gly buffer (pH 8.0), 4.7 g guanidine hydrochloride was added and brought to 10 mL with buffer. After stirring for 25 min, 1 mL was mixed with 5 mL 8 M urea solution and 0.04 mL 4 mg/mL Ellman’s reagents and kept at 25 °C for 30 min. The absorbance was measured at 412 nm using a UV–Vis spectrophotometer (TU-1810, Puxi Instrument Ltd. Co., Beijing, China).

The total sulfhydryl-group (–SH–) was determined as follows: 0.4 mL of the final solution with 2 mL 10 M urea solution and 0.04 mL β-mercaptoethanol were mixed and kept at 25 °C for 1 h. After centrifugation at 2800 g (10 min, 25 °C), the precipitate was washed twice using 5 mL 12% TCA (w/v) dissolved in 6 mL 8 M urea and 0.06 mL Ellman’s reagents and kept at 25 °C for 30 min. The absorbance of the supernatant was determined at 412 nm. The sulfhydryl content for both total and free SH was calculated based on the original Ellman’s extinction coefficient of 13.6 mM^−1^ cm^−1^ for the thiolate chromogen and expressed as mM/g of protein. The disulfide bonds (–SS–) content was obtained using the following calculation: (total SH - total free SH)/2.

### Sensory evaluation

The sensory evaluation was done using 40 untrained volunteers. They were staff and students from the Northeast Agricultural University who were regular cookie consumers from various socioeconomic backgrounds between the ages of 18 and 50. Six samples of freshly-made (within 2 h) cookies (25 °C) were evaluated in each session. Panelists were asked to evaluate the cookies’ color, sweetness, fattiness, crunchiness, hardness and overall liking using a 9-point hedonic scale ranging from 9 (like extremely) to 1 (dislike extremely)^24^ using their best judgment as to how to interprete the various terms. Water was provided to rinse the mouth between evaluations.

### Statistical analysis

All experiments were done in triplicate, and results were expressed as mean ± standard deviation. One-way analysis of variance (ANOVA) with Tukey multiple comparisons test at *P < *0.05 was done to identify significant differences within groups using SPSS software (version 22.0, SPSS Inc., Chicago, IL, USA).

## Results and Discussion

### Proximate composition

The proximate composition of REAE and RSM are shown in Table 1 Despite using different approaches, the moisture, ash and protein in REAE were similar to that in RSM. However, the crude oil content of REAE was approximately 15% lower than the RSM, which was due to the oil extraction yields of EAE being higher (89.5%).

**Table 1:** Proximate composition of REAE and RSM.

Parameters	REAE	Residues from soy milk (RSM)
Moisture (%)	4.7 ± 0.2	4.7 ± 0.1
Ash (%)	4.8 ± 0.6	4.2 ± 0.04
Crude protein (%)	4.2 ± 0.2	22.3 ± 0.4**
Crude fat (%)	3.6 ± 0.1	5.6 ± 0.1*
Crude fiber (%)	83 ± 1	62.2 ± 0.3**
SDF (%)	41 ± 1	40.5 ± 0.2
IDF (%)	41.9 ± 0.3	60.7 ± 0.1**
IDF/SDF	1.0 ± 0.5	1.5 ± 0.3**

Means with * within the same row differ significantly (P < 0.05).

The SDF of REAE was higher than that of the RSM, while the IDF of REAE was lower than the RSM. Similar trends were reported by *Chen, et al*.^25^ who found that the RSM had 58.6% total dietary fiber, including 55.6% IDF and 1.91% SDF. The extrusion-expansion processing increased the SDF content to 30.1%. Previous studies suggested that the IDF decrease could be the result of shear stress during extrusion, which also increased the SDF^26,27^. REAE can be used for water binding, gelling, and structure building, which makes REAE a potentially beneficial food additive.

### *In vitro* digestibility

The percentage of starch in cookies with 0, 10, 20, 30, 40, and 50% REAE was 30, 27, 24, 21, 18, and 15%, respectively. To eliminate the effect of total starch content on the rate of starch hydrolysis, in each test 500 mg of control cookies and 555.6, 625.0, 714.3, 833.3, and 1000.0 mg of cookies with 0, 10, 20, 30, 40, and 50% REAE, respectively, were used.

**Figure 1:**
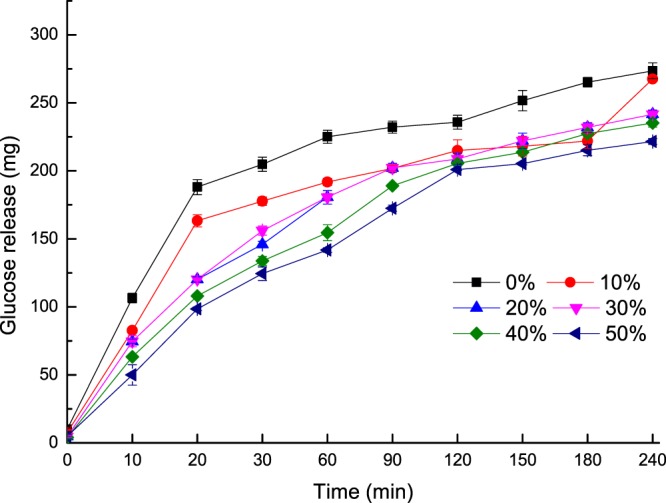
Glucose release from cookies with different levels of REAE after *in vitro* digestion.

**Figure 2:**
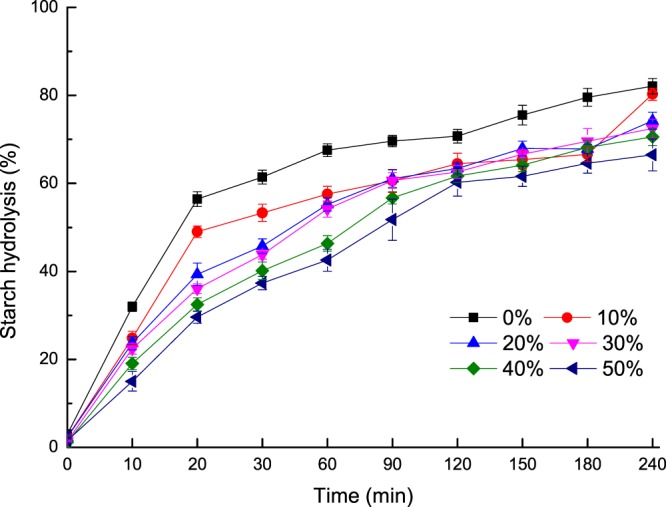
*In vitro* starch hydrolysis (%) in cookies substituted with different levels of REAE.

The glucose released and the starch hydrolysis kinetics of the cookies are summarized in Figs. 1 and 2. As the REAE substitution level increased, the starch digestibility of the cookies was significantly decreased (*P* < 0.05), which is consistent with the previous findings that soluble fiber substitution decreased the rate of starch degradation in bread^28^, and the digestibility decline was obvious in the first 60 min. However, the total starch digestibility of the cookies was not significantly reduced at 240 min (*P* ≥ 0.05). The change in the digestibility may be due to the amount of SDF in cookies as SDF reduces the carbohydrate absorption rate by forming a viscous gel in the small intestine and reducing the postprandial blood glucose response^29^. However, Grundy, *et al*.^30^ suggested that the glycemic effects of food depended on the texture, particle size, starch type, physical entrapment of starch molecules, processing and other ingredients, such as sugar, fat, protein, dietary fiber and anti-nutrient content.

**Figure 3:**
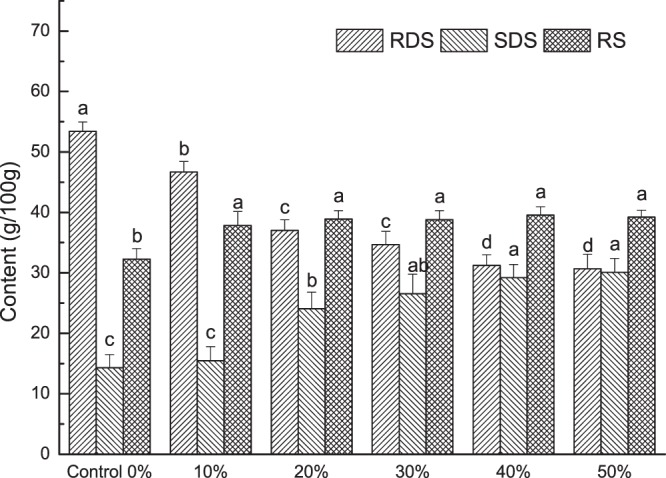
Contents of rapidly digestible starch, slowly digestible starch, and resistant starch in cookies with REAE. Means with different superscript letters within the same column differ significantly (*P* < 0.05). Means with different superscript letters within the same column differ significantly (*P* < 0.05).

The RDS, SDS and RS contents of the cookies are shown in Fig. 3. The SDS and RS significantly increased (*P* < 0.05) as REAE increased, while the RDS content decreased significantly (*P* < 0.05), relatively. The RDS decrease may be due to more moisture in REAE, which might decrease the mechanical rupture of the soy cell wall. In addition, the IDF, which increased the viscosity, may decrease the enzymatic digestion rate of starch^31^. The decrease of RDS indicated that the starch micro-structure was probably almost destroyed during high-temperature baking. These results are in agreement with those of Rosin, *et al*.^32^, who determined that a high dietary fiber concentration may help starch re-associate and further increase the RS content.

**Figure 4:**
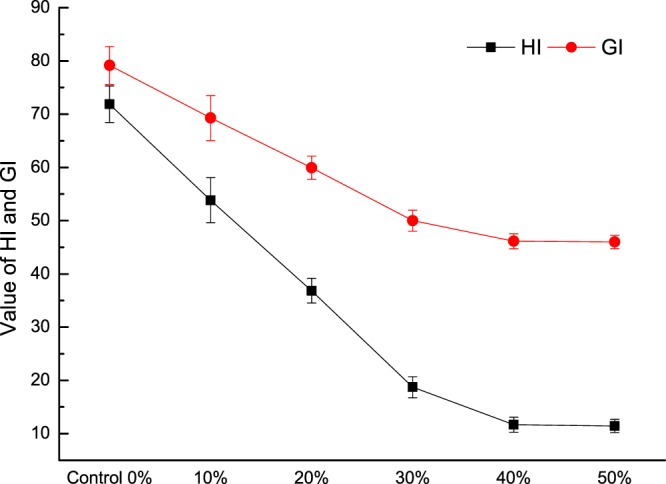
The glycemic index and hydrolysis index values of cookies with different levels of REAE.

Soluble fiber substitution lowered the GI, which is often accompanied by an increase in RS^28^. The HI and GI values are shown in Fig. 4. At higher REAE, the HI and GI significantly decreased (*P* < 0.05). A similar result was reported by Schuchardt, *et al*.^33^ that the glucose release of fiber-enriched cookies was significantly lower than that of wheat cookies and that fiber-enriched cookies had a low-GI. Thus, REAE is suitable for producing a new, slow-digestion food.

### Physical and textural properties of cookies

The physical characteristics and textural properties are important factors when evaluating cookie quality, and the results are shown in Table 2. The cookies’ weight and thickness increased significantly (*P* < 0.05) as REAE increased. However, the cookie diameter did not substantially vary. These changes may be due to higher water retention with a higher fiber content^34^. The hardness, which is an important textural characteristic for cookies increased significantly (*P* < 0.05). The chewiness of the cookies also increased significantly (*P* < 0.05). Previous studies suggested a positive relation between dietary fiber, and hardness and chewiness^35^. According to Cauvain and Young^36^, moisture migrates from the wet core to the drier surface during the baking process, and the subsequent expansions and contractions influence the texture. Thus, REAE showed a positive influence on the texture properties of cookies.

**Table 2:** Physical and textural values of cookies with different levels of REAE.

Sample	Cookies dough			Cookies			
	Hardness (g)	Spring (mm)	Chew (mg)	Weight (%)	Thickness (mm)	Diameter (cm)	Hardness (g)
Control	480 ± 10^e^	1.2 ± 0.2^d^	5.2 ± 0.8^c^	13.2 ± 0.5^e^	4.4 ± 0.3^b^	7.5 ± 0.5^a^	1800 ± 100^c^
10%	550 ± 21^d^	1.5 ± 0.1^bcd^	5.0 ± 0.8^c^	17.4 ± 0.5^d^	4.8 ± 0.5^ab^	7.9 ± 0.2^a^	1700 ± 100^c^
20%	610 ± 10^c^	2.2 ± 0.1^a^	5.8 ± 0.7^c^	20 ± 1^c^	5.0 ± 0.5^ab^	7.6 ± 0.3^a^	1900 ± 400^bc^
30%	673 ± 20^b^	1.9 ± 0.1^ab^	6.0 ± 0.8^c^	22 ± 2^c^	5.1 ± 0.5^a^	7.9 ± 1.0^a^	2100 ± 100^ab^
40%	701 ± 20^ab^	1.7 ± 0.3^bc^	10 ± 1^b^	26 ± 1^b^	5.1 ± 0.3^ab^	7.5 ± 0.1^a^	2330 ± 30^a^
50%	710 ± 19^a^	1.4 ± 0.4 ^cd^	20 ± 2^a^	35 ± 2^a^	5.2 ± 0.2^a^	7.9 ± 0.2^a^	2400 ± 100^a^

Means with different superscript letters within the same column differ significantly (P < 0.05).

### SH/SS content analysis

**Figure 5:**
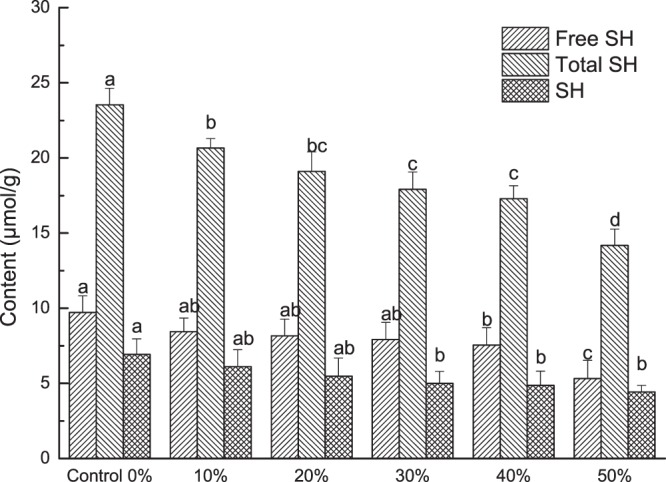
Disulfide (–SS–) and sulfhydryl (–SH–) contents of dough with different levels of REAE. Means with different superscript letters within the same column differ significantly (*P* < 0.05).

The disulfide (SS) bonds and sulfhydryl (SH) groups in the dough with REAE are shown in Fig. 5. With increasing REAE, the SS bonds and total SH groups decreased significantly (*P* < 0.05), while the free SH were not significantly different (*P* ≥ 0.05)^37^. Indicated that a dietary fiber addition led to a reduction in the amount of protein per unit of cookie, which decreased the SS bonds of doughs.

Gluten SH groups significantly influence the structure and functionality of foods by forming SS bonds. SS bonds can lead to protein macromolecules forming network structures, which results in a significant increase in gluten viscosity. Despite the low content of SS bonds, i.e., accounting for 2% of the gluten, SS bonds are important for many protein applications^38^. The SS bonds of gluten protein can be rearranged by oxidation or reduction reactions with the dough additives added during the commercial cookie-making process^39^. In this study there was a linear relationship between the SS bonds (y) and the addition of REAE (x): y = −0.4743 ×  + 7.129, R² = 0.948.

### Sensory evaluation

**Figure 6:**
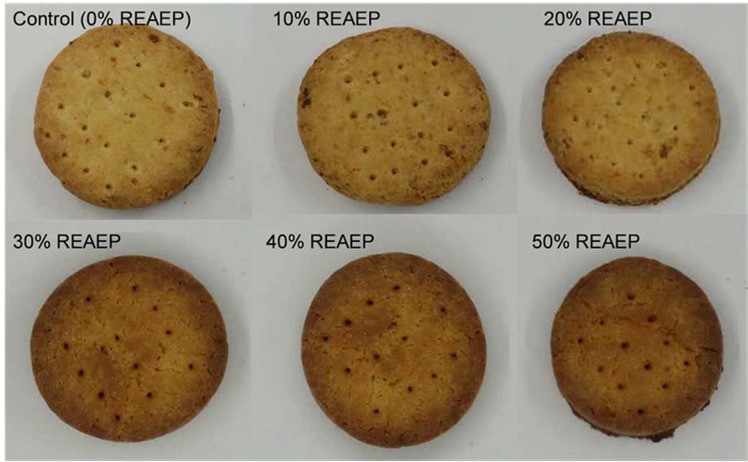
Cookies substituted with different levels of REAE.

The results obtained for the sensory attributes are shown in Table 3. The acceptability of the cookies’ color was significantly decreased (*P* < 0.05) with increasing REAE. The panelists preferred red cookies with a lighter color, and the phenolic compounds in REAE presumably deepen the color (shown in Fig. 6). A similar effect was reported by Jan, *et al*.^24^ with high okara cookies having less desirable color. The sweetness decreased significantly (*P* < 0.05) as REAE increased. The panelists indicated that the cookies had less sugar and fat tastes. In addition, as the substitution levels increased, the cookies were harder. These findings are in agreement with the analytical texture results (Table 2). The 30% REAE cookies were accepted, and the okara addition percentage in wheat products cannot be more than 30% according to Lu, *et al*.^11^ because the excessive dietary fiber dilutes the gluten resulting in deterioration of dough properties.

**Table 3:** Sensory evaluation of cookies with different levels of REAE.

Sample	Color	Sweetness	Fatty	Crunchy	Hardness	Liking	Acceptability
Control	9.2 ± 0.5^a^	7.7 ± 0.3^a^	7.9 ± 1.2^a^	7.9 ± 1.1^a^	5.1 ± 0.7^d^	8.5 ± 1.4^a^	8.7 ± 0.1^b^
10%	7.4 ± 0.5^b^	7.2 ± 0.5^ab^	7.5 ± 1.5^a^	8.5 ± 1.2^a^	6.8 ± 0.7 ^cd^	7.9 ± 1.5^a^	7.8 ± 0.3^c^
20%	7.3 ± 0.8^b^	6.8 ± 0.5^bc^	7.9 ± 1.1^a^	8.8 ± 1.1^a^	7.5 ± 1.1^bc^	7.6 ± 1.6^a^	8.3 ± 0.2^bc^
30%	6.7 ± 0.7^bc^	6.6 ± 0.5^bc^	7.8 ± 1.4^a^	8.8 ± 0.9^a^	8.8 ± 1.3^ab^	7.9 ± 1.0^a^	9.5 ± 0.5^a^
40%	5.3 ± 0.8 ^cd^	6.3 ± 0.6^c^	7.1 ± 0.9^a^	9.1 ± 1.6^a^	9.2 ± 1.1^ab^	7.4 ± 1.6^a^	5.6 ± 0.4^d^
50%	4.7 ± 0.4^d^	6.1 ± 0.2^c^	7.1 ± 1.3^a^	9.1 ± 1.0^a^	9.5 ± 1.5^a^	6.2 ± 1.5^a^	4.8 ± 0.6^e^

Means with different superscript letters within the same column differ significantly (P < 0.05).

## Conclusions

The residues from EAE contained 29.8% soluble dietary fiber and 28.1% insoluble dietary fiber, which was used to replace some of the wheat flour in a cookie formulation. This study indicated that REAE can effectively reduce the starch hydrolysis rate, the content of rapidly digestion starch and the GI. The analytical texture of cookies was acceptable with < 30% REAE and also acceptable to the sensory panel. In addition, the REAE could provide functional benefits. This suggests the potential for using REAE in baked foods as a new source of soybean dietary fiber.
